# Intravenous Prostaglandin F2alpha for the Induction of Labor at a Japanese Perinatal Center

**DOI:** 10.7759/cureus.102192

**Published:** 2026-01-24

**Authors:** Nobuko Yokoyama, Yuria Haruna, Shunji Suzuki

**Affiliations:** 1 Department of Obstetrics &amp; Gynecology, Japanese Red Cross Katsushika Maternity Hospital, Tokyo, JPN

**Keywords:** intravenous prostaglandin f2alpha, japan, labor induction, safety, uterine hyperstimulation

## Abstract

Background: To examine the effect and risks of using intravenous prostaglandin F2alpha, we retrospectively reviewed our cases using intravenous prostaglandin F2alpha for labor induction.

Methods: We reviewed the obstetric records of all nulliparous singleton deliveries at ≥ 22 weeks of gestation from 2004 to 2008 (n = 6,596).

Results: We used intravenous prostaglandin F2alpha to induce labor in 158 nulliparous pregnant women (2.4%) at 39.2 ± 1.4 weeks of gestation under the protocol based on the Japanese clinical practice guidelines. Of these, 64 cases (40.5%) resulted in vaginal delivery, and 44 (27.8%) underwent cesarean delivery during the procedure of intravenous administration of prostaglandin F2alpha. Only one case (0.6%) developed uterine hyperstimulation requiring prompt delivery under the use of prostaglandin F2alpha. There were no cases of serious complications such as fetal death, uterine rupture, or other maternal complications.

Conclusion: Intravenous prostaglandin F2alpha used under the strict protocols seems to be one of the methods that can safely induce labor.

## Introduction

Intravenous prostaglandin F2alpha (dinoprost) has been used for the induction of labor [1,2]; however, its use has seemed to be limited by perceived unacceptable maternal and fetal side effect profiles. In one review of methods of labor induction [1], intravenous prostaglandin F2alpha had a significantly higher rate of uterine hyperstimulation, both with and without fetal heart rate (FHR) changes, than intravenous oxytocin without significant differences in the rate of failing to achieve vaginal delivery. In addition, more maternal side effects were noted in women receiving prostaglandin F2alpha, such as pyrexia, thrombophlebitis, nausea, vomiting, and diarrhea [1,3,4]. The contraindications and restrictions for prostaglandin F2alpha in Japan have remained essentially unchanged over time, and the clinical judgment regarding use has been similar to that for oxytocin when strict monitoring protocols are followed; however, the use of prostaglandin F2alpha has seemed to be restricted in Japan [5,6]. In addition, based on a preliminary review of the literature, there seems to be a paucity of recent literature on intravenous prostaglandin F2alpha.

Therefore, to evaluate the efficacy and risks of intravenous prostaglandin F2alpha for labor induction, we conducted a retrospective review of our cases in which it was administered intravenously.

## Materials and methods

The protocol for this study was approved by the Ethics Committee of the Japanese Red Cross Katsushika Maternity Hospital in Tokyo, Japan. Our institute is one of the major perinatal centers (1,800-2,000 deliveries per year) in Tokyo.

In our hospital, in recent years, although there has been no particular increase in contraindications in the Japanese clinical practice guidelines [5,6], prostaglandin F2alpha has largely fallen out of use. Although it cannot be denied that the relatively specific contraindications for prostaglandin, such as its unsuitability for women with a history of cesarean section, as stated in Japanese guidelines [5,6], may naturally contribute to restraint in the use of prostaglandin F2alpha, in this study, we examined the prostaglandin F2alpha usage cases over the five-year period from 2004 to 2008.

We reviewed the obstetric records of all nulliparous singleton deliveries at ≥ 22 weeks of gestation. In this study, we excluded cases of fetal death, multifetal pregnancy, and serious maternal complications such as severe preeclampsia, and then the inclusion criteria for this study were as follows: nulliparous women with singleton pregnancies whose uterine cervixes had ripened (generally Bishop score ≥ 6) at ≥ 36 weeks of gestation. The method of using prostaglandin F2alpha during the study period in our hospital involved dissolving 3,000 μg in 500 mL of 5% glucose solution or saline (6 μg/mL). The initial dose was 1.5 to 3.0 μg/min (15 to 30 mL/hour) with monitoring of FHR and uterine contractions; the dose was increased by 1.5 to 2.0 μg/min (15 to 20 mL/hour) every ≥30 minutes in cases of unproblematic FHR and uterine contractions, with a maximum dose of 25 μg/min (250 mL/hour) based on the Japanese clinical practice guidelines [5,6].

In this study, side effects of intravenous prostaglandin F2alpha were defined as follows: fetal death during labor, uterine hyperstimulation with non-reassuring fetal status requiring prompt delivery or tocolysis, uterine rupture, pyrexia, thrombophlebitis, nausea, vomiting, and diarrhea [3-7]. In this study, we also examined the maternal age, gestational week at delivery, delivery modes, an Apgar (Appearance, Pulse, Grimace, Activity, Respiration) score of <4 at 1 and 5 minutes, an umbilical artery pH of <7, and total blood loss during delivery.

In this study, data are presented as numbers (%). Statistical analyses were performed with SAS version 8.02 (SAS Institute, Cary, NC, USA). Based on the 4.5% difference in the rate of uterine hyperstimulation associated with FHR changes in the previous review [1], the necessary subject size for the statistical power of the chi-square test with a significance level of < 5% and detection of ≥80% was calculated as 97. In this study, the 95% confidence interval (95% CI) was also calculated.

## Results

We reviewed the obstetric records of all nulliparous singleton deliveries at ≥ 22 weeks of gestation from 2004 to 2008 (n = 6,596). Of these, 158 cases (2.4%) met the inclusion criteria and used intravenous prostaglandin F2alpha for labor induction. The average maternal age and gestational age at prostaglandin F2alpha use were 31.9 ± 5.9 years and 39.2 ± 1.4 weeks of gestation, respectively. During the study period, on the other hand, oxytocin was used in 1,915 cases (29.0%).

Table 1 shows the obstetric outcomes of 158 cases in which intravenous prostaglandin F2alpha was used for labor induction. Among these, 64 cases (40.5%) involved the use of prostaglandin F2alpha due to the failure of effective labor contractions after oxytocin had been administered the previous day. Of these, in 28 cases (43.8%), even with the maximum dose of prostaglandin F2alpha or several hours after initiating it intravenously, effective labor contractions could not be induced, and it was necessary to switch back to oxytocin or other interventions.

**Table 1:** Obstetric outcomes of 158 cases in which intravenous prostaglandin F2alpha was used for labor induction. Data are presented as numbers (percentage).

Variables	Cases used prostaglandin F2alpha
Total number	158
After oxytocin administration	64 (40.5%)
Presence of effective labor contractions	36 (22.8%; /64, 56.3%)
Vaginal delivery	25 (15.8%; /64, 39.1%)
Cesarean delivery	11 (7.0%; /64, 17.2%)
Absence of effective labor contractions	28 (17.8%; /64, 43.8%)
Oxytocin has not been used	94 (59.5%)
Presence of effective labor contractions	72 (45.6%; /94, 76.6%)
Vaginal delivery	39 (24.7%; /94, 41.5%)
Cesarean delivery	33 (20.9%; /94, 10.6%)
Absence of effective labor contractions	22 (13.9%; /94, 23.4%)

In 22 out of the remaining 94 cases (23.4%), even when prostaglandin F2alpha was used up to the maximum dose or several hours after initiating intravenous prostaglandin F2alpha, effective labor contractions were not induced, and it was necessary to switch to oxytocin or other interventions.

From the above, effective labor occurred in a total of 108 cases. Of these, 64 cases (59.3%), 158 (40.5%) resulted in vaginal delivery, and the remaining 44 cases (40.7%), 158 (27.8%) resulted in cesarean delivery.

Of the 106 cases with effective labor contractions, there were nine cases of non-reassuring fetal status requiring cesarean delivery (8.3%), and there was one case of uterine hyperstimulation with non-reassuring fetal status requiring prompt delivery (0.9%, 95% CI 0.02-3.47%; 1/158, 0.6%, 95% CI 0.02-2.44%).

There were no cases of serious complications such as fetal death or uterine rupture. In addition, based on the medical charts, there were no cases of maternal side effects such as pyrexia, thrombophlebitis, nausea, vomiting, or diarrhea. There was one case of neonatal asphyxia with an umbilical artery pH of <7 due to umbilical cord prolapse (0.9%); however, there were no other cases of neonatal asphyxia.

## Discussion

In this study, we used intravenous prostaglandin F2alpha to induce labor in 158 nulliparous pregnant women at 39.2 ± 1.4 weeks of gestation. Of these, 64 cases (40.5%) resulted in vaginal delivery and 44 (27.8%) underwent cesarean delivery; however, only one case (0.6%) seemed to develop uterine hyperstimulation under the use of prostaglandin F2alpha. Moreover, a subset of women who did not respond adequately to oxytocin achieved effective labor following prostaglandin F2alpha administration.

The current results may be contrary to one previous review, indicating the high rates of uterine hyperstimulation with and without FHR changes [1]. In addition, there were no cases with any maternal complications or side effects. These differences may be due to our small study, although the number of subjects was sufficient to meet the study power; however, it cannot be ruled out that these are due to the strict protocol of prostaglandin F2alpha usage in Japan, as mentioned in the Materials and Methods section (maximum dose: 25 μg/min) based on the Japanese clinical practice guidelines [5,6]. In one previous study by Baxi et al. [8], infusions doubled every hour until labor was established (maximum of three doublings), and the maximum dose of intravenous prostaglandin F2alpha was 20 μg/min. In the studies by Vakhariya et al. [9] and Vroman et al. [10], prostaglandin F2alpha was increased to a maximum of 40 μg/min, while in the study by Naismith et al. [11], prostaglandin F2alpha was increased to a maximum of 80 μg/min. In addition, as stated in the methodology of the previous studies in the review, analysis had not been performed according to the individual infusion dosage regime, and it had compromised the heterogeneity of included trials [1]. Therefore, the strict adherence to Japanese protocols may be preventing the side effects of prostaglandin F2alpha suggested in the past reviews [1,5,6]. Searching PubMed and other databases has shown that studies on prostaglandin F2alpha administered intravenously have become scarce since the 21^st^ century [1,12], suggesting that we may be called upon to increase the number of studies and report our findings concerning the clinical usefulness of intravenous prostaglandin F2alpha on labor induction. In 2012, about 83% of major perinatal centers in Japan once used intravenous prostaglandin F2alpha [13,14]. The current situation is unclear; however, the current results support the need for further investigation to confirm the clinical effectiveness and safety of prostaglandin F2alpha.

While the current results may support our continued use of prostaglandin F2alpha with the current methods [5,6], this study may have serious limitations beyond the small sample size. First of all, this study is descriptive; there must be limitations in terms of precision for estimating rare adverse event rates. Secondly, currently in Japan, observation of uterine contractions during labor is primarily conducted using external measurement methods of cardiotocogram [15,16]. Consequently, the diagnosis of uterine hyperstimulation has become subjective, potentially preventing strict comparison with the previous studies. In addition, this is a retrospective study based on the clinical data from 2004 to 2008, representing a period approximately two decades prior to the present. During the intervening years, obstetric monitoring practices, neonatal care capabilities, and institutional protocols may have evolved. Therefore, a large prospective study will be needed to clarify the clinical effectiveness and safety of intravenous prostaglandin F2alpha for the induction of labor.

## Conclusions

We examined the clinical outcomes of labor induction using intravenous prostaglandin F2alpha in 158 nulliparous pregnant women at 39.2 ± 1.4 weeks of gestation at a Japanese perinatal center. By intravenous prostaglandin F2alpha, effective labor occurred in about 68% of them, and about 41% resulted in vaginal delivery. Based on the medical charts, there were no cases with perinatal complications or maternal side effects. Based on the current results, intravenous prostaglandin F2alpha used under the strict protocols seems to be one of the methods that can safely induce labor without causing serious complications for the mothers or fetuses.
